# The National Allergy Centre of Excellence–Addressing Australia's Allergy Burden

**DOI:** 10.1111/cea.70392

**Published:** 2026-07-31

**Authors:** A. Young, J. J. Koplin, D. E. Campbell, J. M. Davies, H. Edmund, T. Jamieson, C. J. Hornung, T. O'Farrell, D. West, E. Kayes, L. A. Gray, R. L. Peters, L. Gamble, K. Gwynne, J. Lee, M. Lucas, C. H. Katelaris, M. D. O'Sullivan, D. J. Palmer, J. Trubiano, S. van Nunen, S. L. Vale, T. Wanandy, K. P. Perrett

**Affiliations:** ^1^ Murdoch Children's Research Institute Parkville Victoria Australia; ^2^ National Allergy Centre of Excellence Parkville Victoria Australia; ^3^ Centre for Food Allergy Research Parkville Victoria Australia; ^4^ The University of Queensland Brisbane Queensland Australia; ^5^ The Children's Hospital at Westmead Westmead New South Wales Australia; ^6^ The University of Sydney Camperdown New South Wales Australia; ^7^ Queensland University of Technology Brisbane Queensland Australia; ^8^ The University of New South Wales Randwick New South Wales Australia; ^9^ Alfred Health Prahran Victoria Australia; ^10^ Austin Health Heidelberg Victoria Australia; ^11^ Monash University Clayton Victoria Australia; ^12^ Sir Charles Gairdner Hospital Nedlands Western Australia Australia; ^13^ Perth Children's Hospital Nedlands Western Australia Australia; ^14^ The University of Western Australia Crawley Western Australia Australia; ^15^ National Allergy Council Sydney New South Wales Australia; ^16^ Western Sydney University Campbelltown New South Wales Australia; ^17^ Campbelltown Hospital Campbelltown New South Wales Australia; ^18^ Australasian Society of Allergy and Clinical Immunology Australia; ^19^ The Kids Research Institute Australia Nedlands Western Australia Australia; ^20^ The University of Melbourne Parkville Victoria Australia; ^21^ Macquarie University Sydney New South Wales Australia; ^22^ The Royal Hobart Hospital Hobart Tasmania Australia; ^23^ The University of Tasmania Hobart Tasmania Australia; ^24^ The Royal Children's Hospital Melbourne Parkville Victoria Australia

## Abstract

Australia has one of the highest rates of food allergy, anaphylaxis hospital admissions and anaphylaxis fatalities worldwide. These trends and sustained advocacy prompted a 2019 Parliamentary Inquiry into Allergies and Anaphylaxis. Among 250 submissions, the Centre for Food Allergy Research called for a national research‐coordinating centre and comprehensive allergy research funding. In response to the inquiry, the Australian Government funded the National Allergy Centre of Excellence (NACE) in 2022, and its partner organisation, the National Allergy Council (NAC), to improve health, save lives and reduce the national burden of allergic disease. Here we describe the establishment and early impacts of the NACE. The NACE was established to build critical national research infrastructure and collaboration to transform consumer‐centred allergy care. Four interconnected Pillars underpin the plan of action: (1) Transforming Allergy Care, codesigning national trials and embedded clinical research to address knowledge and practice gaps; (2) BioRepository and Discovery, developing Australia's first national Allergy BioRepository (ALBI) to support data‐driven discovery; (3) Evidence and Translation, providing living evidence surveillance and synthesis to accelerate translation into policy and practice; and (4) Training and Innovation, building workforce capacity through postgraduate scholarships and fellowships. In just 3 years, the NACE has become a global exemplar, secured further funding and demonstrated excellence in high‐impact research, rapid translation into policy and practice, and improved outcomes for people living with allergic disease. The NACE has united over 500 drug, food, insect and respiratory allergy experts. Over 1500 individuals with allergies are enrolled in five research programs initiated in clinical care, including more than 500 families in a national, standardised peanut oral immunotherapy program (ADAPT) and NACE‐led evaluation study. The ALBI design and build are well‐advanced, and over 5000 academic articles have been assessed through Australia's first drug and food allergy living evidence collections, underpinning clinical guidelines, public health programs and resources. The NACE has been recognised as an European Academy of Allergy and Clinical Immunology Advanced Research Centre and a World Allergy Organisation Center of Excellence. In December 2025, the NACE‐NAC partnership secured further Federal Government funding to continue driving national impact.

## Graphical abstract

Australia's high allergy burden prompted a national response, leading to the establishment of the National Allergy Centre of Excellence (NACE) in 2022. The NACE has advanced research infrastructure, collaboration, and consumer‐centred care through four pillars, achieving global recognition and accelerating translation into policy, practice, and improved health outcomes nationwide.

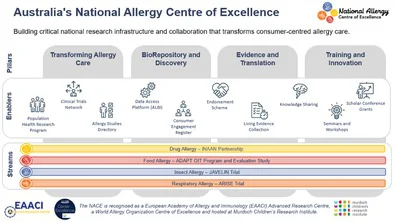

**Abbreviations**
A&AA: Allergy & Anaphylaxis Australia
ACTA: Australian Clinical Trials Alliance
AI: artificial intelligence
ALBI: Allergy BioRepository
AR: allergic rhinitis
ARC: Australian Research Council
ASCIA: Australasian Society of Clinical Immunology and Allergy
CAG: Consumer Advisory Group
CFAR: Centre for Food Allergy Research
CRE: Centre for Research Excellence
CTN: Clinical Trials Network
EAACI ARC: European Academy of Allergy and Clinical Immunology Advanced Research Centre
EMR: Electronic Medical Record
iNAAN: International Network of Antibiotic Allergy Nations
ISAB: International Scientific Advisory Board
JJA: Jack Jumper Ant
LSR: living systematic reviews
MCRI: Murdoch Children's Research Institute
MRFF: Medical Research Future Fund
NAAR: NACE Acute Allergy Registry
NAC: National Allergy Council
NACE: National Allergy Centre of Excellence
NAS: National Allergy Strategy
NHMRC: National Health and Medical Research Council
NSW: New South Wales
OIT: Oral immunotherapy
PADL: Penicillin Allergy Delabelling
QUT: Queensland University of Technology
RADS: Researcher Knowledge, Attitudes and Practices about Health Research Data Sharing in Allergy Study
SNAPR: Setting National Allergy Priorities for Research
UK: United Kingdom
VIT: Venom immunotherapy
WAO: World Allergy Organisation

**Key Message**

- NACE was established as Australia's peak allergy research body in response to a Parliamentary Inquiry.
- NACE Pillars: Transforming Allergy Care, BioRepository and Discovery, Evidence and Translation, Training and Innovation.
- Impacts: strengthened national collaboration, research programs initiated in clinical care, and evidence synthesis underpinning guidelines.

## 1 Introduction

### 1.1 Background to Establishment of the NACE

Australia is considered the ‘allergy capital of the world’ due to the high prevalence of allergic disease. More than eight million Australians are affected, including 11% of infants with clinically confirmed food allergy and almost a quarter of the population reporting allergic rhinitis [1, 2]. Allergies impose a significant physical, emotional and financial burden on individuals, families and carers, and contribute to escalating costs to the healthcare system [3, 4].

In 2019, a bipartisan Australian Federal Parliamentary Inquiry into Allergies and Anaphylaxis was conducted in response to the increasing impact of allergic disease. The Inquiry examined the causes and assessed the management and treatment of allergies and anaphylaxis to identify opportunities for improved support and explore the need for a nationally consistent approach. Over 250 submissions were received from government agencies, peak industry and professional organisations, community support and patient advocacy groups, health providers, research organisations and universities, and the general public. Among these was a submission from the Centre for Food Allergy Research (CFAR), a national collaboration of food allergy researchers and the National Health and Medical Research Council (NHMRC) Centre for Research Excellence (CRE, GNT 1134812). CFAR advocated for funding across all aspects of allergic disease research, not just food allergy, and for the establishment of a national centre.

The Inquiry's final report, *Walking the Allergy Tightrope: Addressing the Rise of Allergies and Anaphylaxis in Australia*, presented 24 recommendations to advance the prevention, intervention, management and treatment of allergic disease via a national plan of action and further investment to address this continuing public health burden [5]. The report emphasised the importance of Australia's world‐leading allergy research in describing the prevalence of allergy in Australia and uncovering its causes—knowledge essential for developing strategies to halt and reverse the rising trend. It also highlighted the success of CFAR, funded by the NHMRC since 2013 and accredited in 2020 as a World Allergy Organisation (WAO) Center of Excellence, in supporting emerging researchers, advancing knowledge generation and strengthening Australia's reputation as a global leader in innovative allergy research.

Additionally, the report acknowledged the productive partnership between CFAR and the former National Allergy Strategy (NAS), a collaboration between the peak professional body for clinical immunology and allergy in Australia and New Zealand, the Australasian Society of Clinical Immunology and Allergy (ASCIA) and national patient support organisation Allergy & Anaphylaxis Australia (A&AA) [6]. This partnership brought researchers, clinicians, and consumers together, enabling research evidence to be rapidly translated into public health policy, clinical guidelines, and community resources.

In response to the report, the Australian Government announced a $26.9 million investment in the March 2022 Federal Budget to establish the National Allergy Centre of Excellence (NACE) and to transition the existing NAS into the National Allergy Council (NAC). The NACE, as a new body, encompassing drug, food, insect and respiratory allergies, was tasked with developing critical research infrastructure and clinical innovation to maintain Australia's global leadership and strengthen evidence‐based management of allergic disease. The NAC, continuing as a partnership between ASCIA and A&AA, delivers evidence‐based public health initiatives, education resources and training programs. Working alongside NAC, the NACE ensures allergy programs and clinical guidelines stay laser‐focused on the latest evidence. Together, the NACE and NAC address the report's recommendations, tackle the escalating health and economic impacts of allergic disease, and ultimately improve outcomes for 8.2 million Australians living with allergic disease (Figure 1).

**FIGURE 1:**
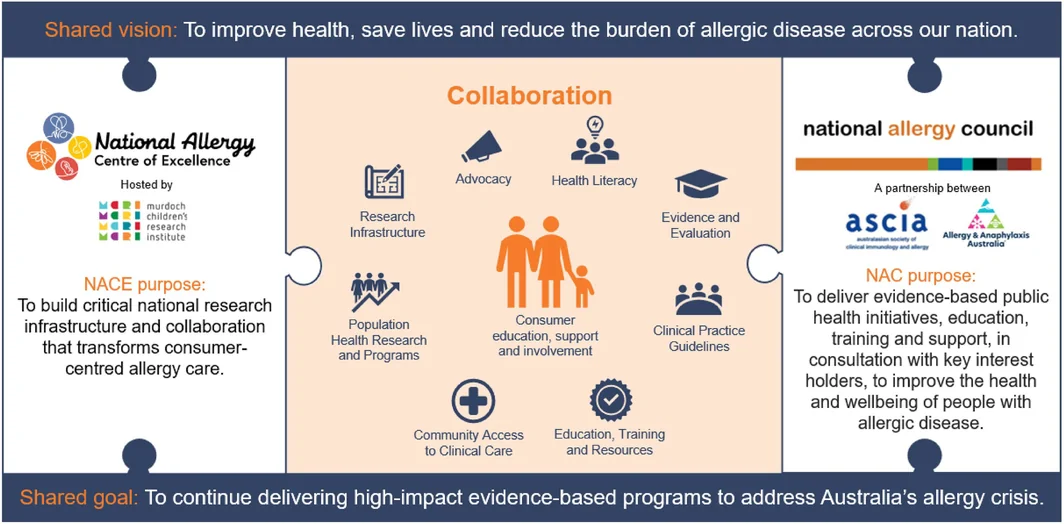
Partnership of the National Allergy Centre of Excellence and the National Allergy Council.

## 2 Establishment and Structure of the NACE

### 2.1 NACE Vision

The NACE established a world‐first model for a national allergy research body, uniting Australia's leading drug, food, insect and respiratory allergy experts. As Australia's peak allergy research body, it drives clinical innovation, builds national research infrastructure, fosters collaboration and supports the delivery of consumer‐centred allergy care. The NACE accelerates high‐quality research and provides consumers, clinicians and policy makers with access to the latest evidence‐based research findings and clinical trials. Sharing a common vision with its partner organisation the NAC, the NACE aims to improve health, save lives and reduce the burden of allergic disease, supporting the delivery of Australia's national plan of action to address the allergy burden.

### 2.2 Allergy Research in the Australian Context

In Australia (population 27 million in 2025), allergy research occurs in universities, independent medical research institutes, and hospitals across eight states and territories. Membership of the NACE is open to all allergy researchers nationally, and members come from 73 universities/institutes/hospitals and include members from all Australian states and territories.

### 2.3 NACE Program Structure

The NACE program is divided into four interconnected Pillars designed to maximise outcomes and establish the systems, processes and infrastructure to support allergy research across the four allergy Streams—drug, food, insect and respiratory (Figure 2).

1. *Transforming Allergy Care*: pinpointing critical knowledge and care gaps and co‐designing tailored national studies and embedded clinical programs with consumers to fast‐track evidence into practice within a learning health system framework, to rapidly transform care.
2. *BioRepository and Discovery*: establishing a platform to integrate fragmented allergy datasets into a national research data asset that will accelerate discovery and improve health.
3. *Evidence and Translation*: establishing living systematic and scoping reviews to connect evidence and practice faster than ever before and describe the evolving spectrum of allergic disease.
4. *Training and Innovation*: supporting emerging allergy researchers through competitive postgraduate scholarships, fellowships and mentoring.

**FIGURE 2:**
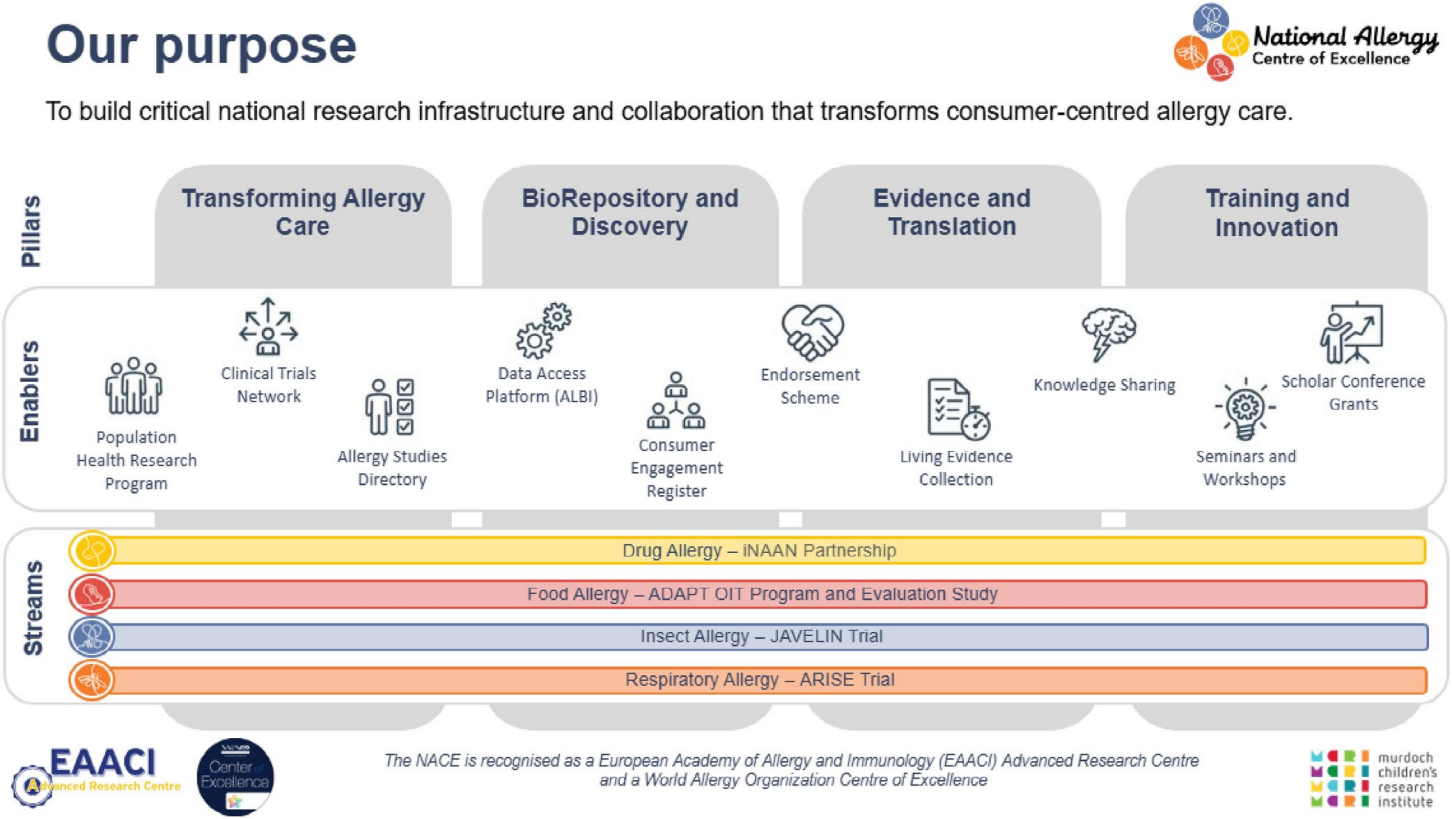
Organisation of activity pillars and streams of the National Allergy Centre of Excellence.

The NACE also delivers a series of strategic cross‐cutting initiatives that enhance and connect all four Pillars.

### 2.4 Governance and Leadership

A comprehensive governance structure guides the design and delivery of NACE activities. The foundational NACE Executive Committee, comprising the NACE Director and Pillar Leads, provides strategic oversight and monitors program delivery and risk. Each allergy Stream is supported by an elected Stream Advisory Group of 10–15 members with two Co‐chairs. These groups meet three to four times per year to provide direct input into Pillar activities and ensure Stream‐specific needs inform national research priorities. The NACE Leadership Group brings together the Co‐chairs of the four Stream Advisory Groups, the NACE Executive, the Co‐chairs of the NACE Consumer Advisory Group and a NAC representative. This group provides national‐level strategic input across NACE initiatives and ensures alignment with its broader mission.

Membership across the four Streams is open to allergy researchers and healthcare professionals nationwide, providing a sustainable framework for collaboration. Current membership is more than 500 individuals, with many participating in multiple Streams. Members include postgraduate students, early‐ and mid‐career researchers, and senior leaders across disciplines, including clinician‐researchers, laboratory scientists, epidemiologists, statisticians, knowledge translation specialists, clinical triallists, and research assistants.

To ensure global relevance, alignment with best practice and international collaboration, the NACE has established an International Scientific Advisory Board (ISAB). Co‐chaired by the NACE Director and an international allergy clinician‐researcher, the ISAB includes seven experts from North America, the UK, Europe and Australia who advise on scientific strategy, global opportunities and international engagement.

### 2.5 Consumers Driving Allergy Research

#### 2.5.1 Consumer Advisory Group

The NACE recognises that meaningful consumer involvement is essential to its success. Consumers bring lived experience, insight and diverse perspectives that shape decisions on research activities and infrastructure projects. As the beneficiaries of advances in allergy care, consumers have a strong interest in ensuring that high‐quality research is translated into improved policy, guidelines and clinical practice.

In its first year, the NACE launched a national Expression of Interest process to form its inaugural Consumer Advisory Group (CAG) [7]. Following a rigorous interview process, eight members and two Co‐chairs were appointed. They represent Australians living with and caring for individuals with drug, food, insect and respiratory allergies. The CAG's role is to ensure the consumer voice is truly embedded in Australian allergy research and programs, including priority setting, design, implementation, evaluation and advocacy. Members received training in consumer involvement in research, and the NACE implemented a remuneration policy consistent with industry best practice to recognise their contribution.

The CAG meets quarterly and provides input across key NACE initiatives, including Stream activities and postgraduate scholar projects. Members also contribute on an ad‐hoc basis when specialised advice is required. The two Co‐chairs serve on the NACE Leadership Group, ensuring consistent representation of consumer priorities in strategic decision‐making.

#### 2.5.2 Consumer Engagement Register

In addition to the CAG, the NACE established a Consumer Engagement Register, which includes over 100 people living with, or caring for someone with, allergies who wish to share their lived experience. Members partner with researchers to ensure the consumer voice is embedded across research and to meet expectations set by Australia's key health and medical research funders. To date, Register members have contributed to more than 30 research projects by reviewing study materials and participating in targeted workshops and focus groups. As consumer contributions become increasingly central to high‐quality research, demand for the Register members' involvement is expected to grow.

## 3 Impacts and Learnings from the NACE to Date

### 3.1 Pillar 1: Transforming Allergy Care

Better prevention, diagnosis, and treatment strategies are urgently needed to counter Australia's allergy burden. This Pillar addresses critical knowledge and care gaps, and co‐designs national studies and improvement science programs with consumers (Figure 3). To date, over 1500 individuals with allergies have been enrolled in five NACE research programs initiated in clinical care, as described below. This includes over 560 families enrolled in the ADAPT national, standardised peanut oral immunotherapy (OIT) program in 10 paediatric hospitals across five Australian states.

**FIGURE 3:**
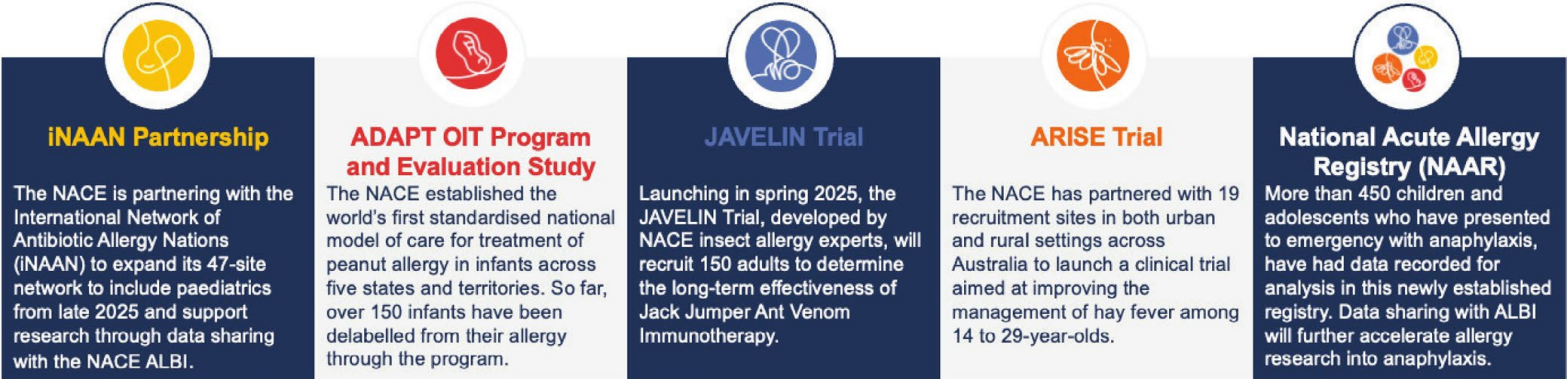
NACE Pillar 1 Transforming Allergy Care studies.

#### 3.1.1 The ADAPT OIT Program and Evaluation Study—A Paradigm Shift in Peanut Allergy Treatment in Australia

The ADAPT OIT Program is a partnership between the NACE and 10 paediatric public hospitals across five Australian states who deliver the clinical program. It is the world's first standardised national model of care for infants with peanut allergy [8] and the first disease modifying treatment of food allergy available within the Australian public hospital setting.

##### 3.1.1.1 ADAPT Methodology

Driven by strong consumer advocacy, it provides free, structured OIT for eligible infants under 12 months of age. Infants undergo a threshold food challenge or microdose escalation to confirm peanut allergy prior to treatment. A concurrent evaluation study was granted a waiver of informed consent by the RCH Ethics Committee (HREC/103759/RCHM‐2024) as the research involved no collection of patient‐identifiable data. Site‐specific governance approval was obtained at each site. It uses a learning health system framework to analyse data in a de‐identifiable aggregate form to assess feasibility, effectiveness, and parent‐reported outcomes in real‐time, supporting continual program improvements. Expansion to other sites, including in regional areas, and introducing OIT to other allergens and age ranges is being explored to support scalability and equitable access to this treatment.

#### 3.1.2 JAVELIN Trial—Optimising Jack Jumper Ant Venom Allergy Management

Insect allergy remains a leading cause of fatal anaphylaxis in Australia [9], with unique allergens like the Jack Jumper Ant (JJA) presenting challenges, especially for regional and rural communities. JJA venom immunotherapy (VIT) is available and currently offered in three centres, but there is limited evidence on long‐term effectiveness and quality‐of‐life outcomes.

##### 3.1.2.1 JAVELIN Methodology

The JAVELIN Trial (NCT06935890; HREC/111294/RCHM‐2025), a partnership between these centres and the NACE, will obtain written informed consent and recruit 100 adults post completion of JJA VIT program for supervised sting challenges and longitudinal surveys over 3 years. Findings will refine treatment pathways, guide health service delivery and support advocacy for Pharmaceutical Benefits Scheme listing of JJA VIT to improve access, affordability and equity of care.

#### 3.1.3 ARISE Trial—Improving the Lives of Young Australians With Hay Fever

Hay fever or allergic rhinitis (AR) affects nearly 24% of Australians [2], contributing to asthma exacerbations, sleep apnoea, reduced productivity, and poorer mental health if inadequately controlled. Despite effective treatments, adherence is low among those with moderate‐to‐severe AR, limiting clinical outcomes.

##### 3.1.3.1 ARISE Methodology

The NACE ARISE Trial (NCT06686446; HREC/110157/RCHM‐2024) was designed to test whether a targeted education package—using existing consumer‐facing resources from NAC, ASCIA and A&AA—improves symptoms and patient‐reported outcomes compared with standard care. The trial aimed to obtain verbal informed consent via telehealth and recruit 194 Australians aged 14–29 through content marketing and referrals. ARISE sought to enhance AR control, reduce the need for immunotherapy, and improve quality of life, with potential for national implementation if effective.

#### 3.1.4 iNAAN Kids Study—Antibiotic Allergy Delabelling in Children

Around 18% of hospitalised Australians report antibiotic allergy, and around 5% of children are impacted—a rate that increases with age—yet most labels are inaccurate, leading to suboptimal antibiotic prescribing [10, 11]. The adult‐focused International Network of Antibiotic Allergy Nations (iNAAN) demonstrated safe and efficient delabelling using the PEN‐FAST risk assessment tool and direct oral challenge (DOC) with more than 47 sites globally using this protocol [12].

##### 3.1.4.1 iNAAN Kids Methodology

To extend these benefits to children, the NACE partnered with iNAAN to establish iNAAN–Kids which was granted a waiver of informed consent by the RCH Ethics Committee (HREC/119109/RCHM‐2025) as the research involved no collection of patient‐identifiable data. It uses modified risk assessment tools and DOC protocols to prevent the persistence of inaccurate labels. Initially piloted in Australia, iNAAN Kids could be expanded internationally. De‐identified Australian data will feed into the NACE Allergy BioRepository to support future research. The NACE will also explore expanding iNAAN to include cephalosporin allergy delabelling in adults.

#### 3.1.5 The NACE Acute Allergy Registry (NAAR) – Improving Anaphylaxis Care in the Emergency Department

Anaphylaxis rates continue to rise in Australia. Progress in research has been hindered by inconsistent hospital coding, variable care settings, and difficulties capturing structured clinical data [13, 14].

##### 3.1.5.1 NAAR Methodology

The NACE, in partnership with The Royal Children's Hospital Melbourne, developed the NAAR which was granted a waiver of informed consent by the RCH Ethics Committee (HREC/102882/RCHM‐2023) as the research involved no collection of patient‐identifiable data. The NAAR stores information on the triggers, symptoms and clinical management of acute allergy and anaphylaxis systematically collected through the Electronic Medical Record (EMR). Continual evaluation of the NAAR data, including use of a novel Severity Grading Matrix, will inform data‐driven improvements in the care of paediatric patients with anaphylaxis, with plans to expand to the adult emergency department setting. Insights from the NAAR will also inform the development of a National Anaphylaxis Reporting System, one of the NAC's flagship initiatives.

#### 3.1.6 Lessons Learnt in the Development and Implementation of Pillar 1 Research Programs

##### 3.1.6.1 Research Programs Embedded in Clinical Care

This pillar aims to embed research programs in clinical care under a learning health system framework, ideally embedded in EMRs. This approach posed unexpected challenges due to differences in EMR platforms across sites, even for sites using the same EMR platform with variable types of data collected, terminology, and coding practices. This poses challenges with establishing studies truly embedded in clinical care and to be successful requires extensive input from EMR technical experts to support back‐end development to structure the data collection and harmonise datasets across sites.

##### 3.1.6.2 Learnings From the ADAPT Program

Key factors in the success of the ADAPT OIT program include its collaborative approach to design, with paediatric tertiary hospitals from five states involved in the initial working group; significant consumer involvement from the outset; and development of nationally standardised patient education resources and dosing guidelines [8]. Capacity to support the ADAPT OIT clinical program is dependent on public hospital site resources and funding, which, without substantive additional investment, has posed challenges to implementation at some sites.

##### 3.1.6.3 Learnings From the ARISE Trial

The trial closed in early 2026 due to insufficient recruitment despite protocol amendments and a range of recruitment strategies including social and mainstream media to raise interest and awareness, highlighting the difficulties in recruiting this age group and the importance of meaningful consumer engagement at every stage to deeply understand the behaviours and needs of the target population for successful trial conduct.

### 3.2 Pillar 2: Biorepository and Discovery

While Australia is a global leader in allergy research, national data remains fragmented, creating barriers to consolidating information and generating new insights efficiently. To address this, allergy and digital health experts in Pillar 2 are developing the NACE Allergy BioRepository (ALBI) (HREC/107531/RCHM‐2024), an advanced platform designed to securely integrate allergy data and biospecimen information from NACE‐led and endorsed studies [15].

#### 3.2.1 ALBI Methodology

ALBI is guided by the FAIR principles to ensure data is Findable, Accessible, Interoperable and Reusable [16], as well as the Five SAFEs Framework which ensures safe people, projects, settings, data, and outputs [17]. These frameworks shape ALBI's architecture and governance to enable secure, ethical, and high‐quality data use. The NACE has developed detailed requirements, solutions and data architecture, documentation and coding standards that cover demographic data, consent items, privacy preservation, data quality and study metadata. These elements underpin ALBI's data management framework, business rules and data workflows.

A key feature of ALBI is the use of advanced unidirectional pseudo‐anonymisation and encryption, enabling secure data transfer and storage while preserving participant privacy. This approach also allows for potential data linkage in future with external national datasets, broadening the platform's research value. To enhance interoperability, common data modules, such as standardised demographic and family allergy history questionnaires, will enable harmonised data capture (Figure 4). A Scientific Access Framework will govern how researchers request and access integrated ALBI data for future HREC‐approved projects, supporting hypothesis‐driven and big data analyses. Technical design is complete with the ALBI launch planned for mid‐2026 as a national research asset that will accelerate discovery that delivers insights to improve health outcomes.

**FIGURE 4:**
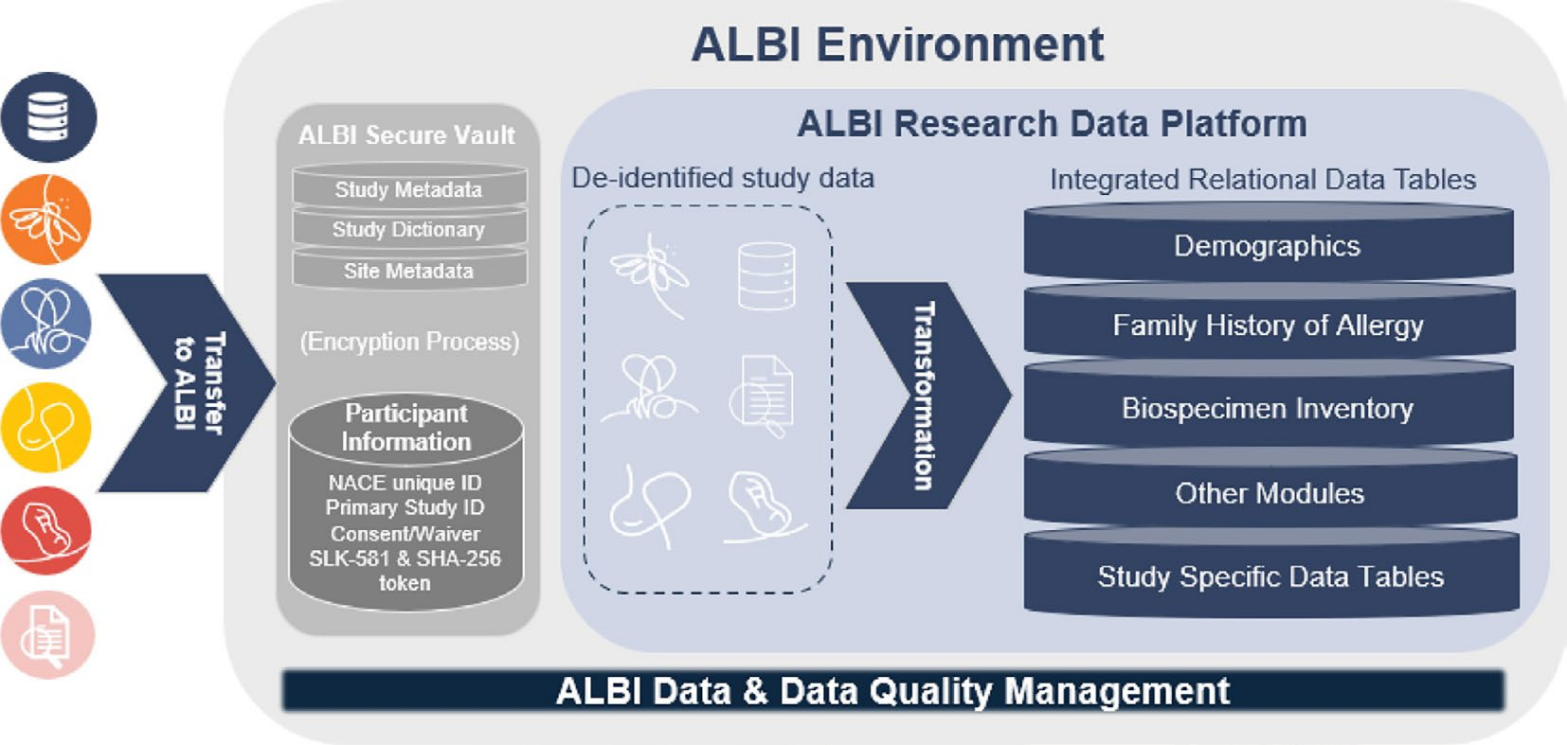
Schema of integration and storage of data in ALBI for data transferred from allergy studies or registries for further allergy research.

To ensure ALBI aligns with end user needs, the NACE is undertaking the Researcher Knowledge, Attitudes and Practices about Health Research Data Sharing in Allergy (RADS) Study (Queensland University of Technology HREC# 9238). RADS includes two components: an analysis of allergy and other studies registered in the Australian New Zealand Clinical Trials Registry, and a national survey of researchers whose work involves people living with allergic conditions. The survey captures knowledge of data sharing guidelines, current and past data‐sharing experiences, perceived challenges and enablers, and attitudes toward contributing to and accessing ALBI. Insights will directly inform ALBI's data access and governance processes.

In parallel, Pillar 2 partnered with the Melbourne Children's Lifecourse Initiative to create the NACE Allergy Studies Metadata Platform [18]. Launched in 2024, the platform catalogues completed and active Australian allergy studies to improve visibility and support secondary use of data and biospecimens. It currently includes 15 studies and is expected to expand as awareness and engagement grow.

#### 3.2.2 Learnings From the ALBI Project

Challenges to be overcome in delivering these types of data repositories include the technical design and build of the repository to receive and integrate allergy study data, ensuring appropriate consent from study participants to enable reuse of data. The current context of limited experience with and perceived barriers to data sharing among researchers, and unstandardised allergy study data, appears to be both hurdles and motivators for development of ALBI. Opportunities include the structured use of AI to streamline big data analysis in the future if these hurdles can be overcome.

### 3.3 Pillar 3: Evidence and Translation

Allergy research is advancing rapidly, making it essential that evidence surveillance and synthesis keep pace with emerging data. Systematic reviews are the gold standard for summarising research, supporting evidence‐based decision‐making and identifying gaps. However, traditional reviews are static and quickly become outdated. Instead, Living Systematic Reviews (LSRs) continuously incorporate new evidence, making them critical in the fast‐moving field of allergy research.

Through this Pillar, the NACE is developing a Living Evidence Collection across drug, food, insect, and respiratory allergy. Using extensive stakeholder input to prioritise topics and guide methods, the NACE is embedding living evidence in guideline development, healthcare delivery, research prioritisation, and policy. This ensures allergy care in Australia is consistently underpinned by the most up‐to‐date and reliable global evidence.

To date, the NACE has established four living reviews, one each in drug, food, insect, and respiratory allergy. Evidence synthesis by the NACE has underpinned three ASCIA guidelines across food allergy prevention and penicillin allergy delabelling, as described below.

#### 3.3.1 Drug Allergy

Inaccurate antibiotic allergy labels pose a serious risk to antimicrobial stewardship and increase healthcare costs through the avoidance of low‐cost first‐line antibiotics. To address this, the NACE, NAC, ASCIA and A&AA have worked in partnership to develop new ASCIA Penicillin Allergy Delabelling (PADL) Guidelines for adults. The NACE conducted a rapid overview of reviews [19], followed by a NAC‐led Delphi process, which directly underpinned these guidelines and informed a national antibiotic allergy communications strategy and program. The NACE Living Overview of Reviews on PADL [20] will be continuously updated and guide future updates to ASCIA Guidelines, resources and training, as well as A&AA's consumer resources. The first evidence update has already informed the forthcoming ASCIA Paediatric PADL Guidelines, developed through a similar NAC‐led Delphi process and incorporated into the NAC's communication strategy targeting children and families.

#### 3.3.2 Food Allergy

The NACE and CFAR partnered with Germany's HELICAP team to coordinate LSRs on early childhood allergy prevention. An initial overview on complementary feeding and allergy prevention identified early introduction of specific foods as beneficial while highlighting key evidence gaps [21]. This work directly supported the first major update since 2016 to the ASCIA Guideline: Infant Feeding for Food Allergy Prevention [22] and the NAC's *Nip Allergies in the Bub* website [23] and resources. Updates were informed through evidence summaries, a series of key stakeholder meetings involving ASCIA, NAC, NACE, CFAR and A&AA, and a NAC‐led Delphi process. A priority NACE LSR on Vitamin D for the Prevention of Food Allergy and Eczema was launched [24], with its fourth update now completed.

#### 3.3.3 Insect Allergy

Tick‐induced allergies, including mammalian meat allergy and tick anaphylaxis, are increasing in Australia. Following recommendations from the 2025 Senate Inquiry into the access to diagnosis and treatment for tick‐borne diseases [25], a NACE postgraduate scholar led a scoping review on allergic and inflammatory sequelae of tick bites [26]. In partnership with the Pillar 3 team, this will transition into a LSR to synthesise emerging evidence to address knowledge gaps and inform resources to improve clinical and consumer awareness.

#### 3.3.4 Respiratory Allergy

Aeroallergen immunotherapy offers disease‐modifying benefits but faces challenges with cost, adherence and product variability. In the Respiratory Allergy Stream, the Pillar 3 team will transform a NACE PhD candidate's systematic review on the cost‐effectiveness of immunotherapy [27] into an LSR to provide ongoing synthesis of data to guide clinical and policy decisions and inform future research.

#### 3.3.5 Shared Care in Allergy

Improving equitable and timely access to allergy care is a national priority. In partnership with the NAC, the NACE conducted a scoping review of international allergy shared care models [28]. This evidence informed national discussions and workshops led by the NAC, ASCIA and A&AA with healthcare professionals and people living with allergies. These activities shaped the Shared Care for Allergy Implementation Roadmap [29], which outlines opportunities to improve access to high‐quality allergy services and strengthen collaborative models of care.

##### 3.3.5.1 Learnings From the Evidence and Translation Pillar

Ensuring that evidence synthesis is both high quality and useful for informing public health and clinical practice guidelines requires strong partnerships between researchers, clinicians and consumers. In NACE, this has been achieved through strong partnerships with NAC, ASCIA and A&AA. The success of this pillar's activities relies on extensive stakeholder engagement in the early stages of designing reviews to establish clearly defined research questions, and flexibility on the part of researchers and methodologists—applying a combination of more traditional systematic review methodology, rapid reviews, scoping reviews and overviews of reviews as required.

### 3.4 Pillar 4: Training and Innovation

Training the next generation of allergy researchers is critical to ensure sustained workforce capacity. The NACE is increasing the number and diversity of allergy researchers with expertise across all areas, including immunology, data science, clinical trials, epidemiology, health economics and implementation science. This drives novel approaches to allergy prevention, diagnosis and treatment and maintains Australia's position at the forefront of international allergy research and translation.

In 2023, the NACE identified future leaders in allergy research, awarding seven Postgraduate Scholarships and 11 Postdoctoral Fellowships. These 18 researchers—working across basic science, epidemiology and clinical research—contribute to all four allergy Streams and advance targeted Pillar activities. Scholars and fellows receive supervision from senior NACE researchers and benefit from a rich program of symposiums, in‐person and virtual events and training days that offer high‐calibre educational, networking and mentoring opportunities.

All NACE‐funded scholars must complete a systematic or scoping review as part of their project, with Pillar 3 providing expert guidance and training. Scholar Conference Grants are available to support the showcasing of NACE‐led allergy research nationally and internationally and foster collaboration. To date the scholars and postdoctoral fellows supported by NACE have led 26 publications.

### 3.5 NACE Cross‐Cutting Platform of Tools and Resources

#### 3.5.1 Knowledge Sharing

A key priority for the NACE is sharing activities and new knowledge with policymakers, clinicians, industry, the public, donors and collaborators. Supported by the NACE Communications Manager, NACE‐led media, social media and online content have reached over 1.5 billion people globally. The Allergy Studies Directory, originally established by CFAR, was expanded across all Streams and now includes over 75 allergy studies—many actively recruiting—with more than 2300 subscribers receiving alerts when new studies are listed [30]. Alongside this, the Publications Directory promotes manuscripts by NACE leaders and scholars, with > 150 allergy publications in the past 3 years. This outreach has raised global awareness of Australian allergy research, positioned the NACE as the country's peak allergy research body, and established its members as key opinion leaders sought after by media and peers.

#### 3.5.2 Clinical Trials Network

Allergy clinical trials are a core component of research in Australia, and NACE researchers lead or contribute to many pivotal investigator‐initiated and industry‐sponsored clinical trials in allergy. To support this work, the NACE Clinical Trials Network (CTN) was established and has been granted Associate Membership of the Australian Clinical Trials Alliance (ACTA). The NACE CTN aims to accelerate the start‐up and conduct of national allergy trials. This ensures timely access to innovative prevention and treatment options for individuals and families and supports collaboration between triallists and industry sponsors. A national scoping exercise identified priority resources for Australian allergy triallists, leading to the creation of a resource hub, leveraging existing tools—including templates and standard operating procedures available through ACTA and the Australian Clinical Trials Education Centre—alongside NACE‐developed resources.

#### 3.5.3 Population Health Research Program

Aligned with *Walking the Allergy Tightrope*, the NACE Population Health Research Program analyses national data to better understand allergy prevalence and burden, particularly among underrepresented groups, including First Nations people and those in rural or lower socioeconomic areas. Analysis of over 17,000 households in the National Health Survey found self‐reported rates of 23.9% for allergic rhinitis, 7% for food allergy, and 5.2% for drug allergy, with lower rates in disadvantaged groups [2]. The Program is also using Victoria's School Entrance Health Questionnaire data to determine allergy prevalence in children beginning school, including those from underrepresented groups. To align future research with community needs, the NACE is leading the Setting National Allergy Priorities for Research project (SNAPR), identifying priority areas determined jointly by health care professionals and those living with or caring for someone with allergies. This work will guide funding toward research most likely to improve outcomes for Australians with allergic disease.

#### 3.5.4 Policy and Advocacy

The NACE contributes evidence and expertise to government inquiries and consultations. In 2024, NACE insect allergy experts made submissions and appeared at the public hearing of the Parliamentary Inquiry into Red Imported Fire Ants [31], subsequently publishing a narrative review [32]. That year, the NACE responded to a government consultation advocating for allergic diseases to be recognised as chronic conditions. In 2025, the NACE made submissions to the Senate Inquiry into access to diagnosis and treatment for tick‐borne diseases [33] and supported ASCIA's application to the Medical Services Advisory Committee for a new Medicare Benefits Schedule item for supervised oral food challenges. The NACE also endorsed the updated Australia and New Zealand consensus statement on best‐practice food allergen management [34].

Strategic relationships with the Australian Government will continue to be strengthened with the establishment in late 2025 of the Parliamentary Friends of Allergy which provides a non‐partisan forum for 20 parliamentarians to meet and interact with the NACE, NAC, ASCIA and A&AA on matters relating to allergy and anaphylaxis across Australia.

#### 3.5.5 Endorsement Scheme

The NACE Research Endorsement Scheme supports NACE members seeking formal endorsement of their allergy studies. These studies must demonstrate scientific quality, feasibility, potential for translation of results into clinical practice or policy, and alignment with NACE's vision. The scheme promotes collaboration, focuses attention on priority allergy issues, and upholds high standards in study design, conduct, and reporting.

### 3.6 International Context and Global Recognition of the NACE

In recognition of NACE's early impacts, NACE was awarded an inaugural European Academy of Allergy and Clinical Immunology (EAACI) Advanced Research Centre (ARC) status as part of the EAACI Quality Centres initiative in June 2025. This recognition highlights leadership in allergy research and contribution to national and international collaboration and innovation. ARC centres gain access to exclusive EAACI opportunities, including fellowships, mentorships, task force participation, congress attendance, and travel grants, supporting the development of the next generation of allergy researchers.

In March 2026, NACE received further endorsement that it meets international standards of excellence as a World Allergy Organisation Center of Excellence. This recognition will enhance global collaboration opportunities and strengthen NACE's ability to influence research, education and clinical practice worldwide.

Similar initiatives, actions and programs to improve allergy management also exist internationally. The Finnish Allergy Programme, a 10‐year national program to improve prevention and management of allergic diseases and asthma, was implemented in Finland between 2008 and 2018. Reported outcomes include a “levelling off” of the prevalence of allergic diseases and asthma, a reduction in asthma symptoms and food allergy diets in day care and schools, and a reduction in the direct and indirect costs of asthma and allergic diseases [35]. In the UK, the development of a National Allergy Strategy is currently underway, with contributions from the British Society for Allergy and Clinical Immunology, Allergy UK, Anaphylaxis UK and the Natasha Allergy Research Foundation [36]. In the United States, the National Institute of Allergy and Infectious Diseases is a large federal model which aims to support innovative research to address fundamental studies in immunology and allergic and autoimmune diseases to improve patient outcomes [37].

### 3.7 Lessons Learnt From the Establishment and First Years of the NACE

In just under 4 years, the NACE has demonstrated excellence in generating high‐impact research and rapidly translating evidence into policy and practice and has secured continued funding to June 2028. A recent editorial in Clinical and Experimental Allergy recognised that the NACE's achievements “highlight the value of a coordinated, nationally funded allergy research programme” [37]. Built on a strong foundation of research excellence and existing collaborations in allergy research in Australia, the widespread involvement of researchers and clinicians nationally in the NACE as well as the partnership with the NAC, ASCIA and A&AA, and strong links to the Australian Government are key factors driving this success. On an operational level, it is important for the success of projects to have specific content expert project leads supported by a project manager to deliver the operations. Importantly, meaningful involvement of consumers is essential to the success of NACE in achieving the vision of improving health, saving lives and reducing the burden of allergic disease.

### 3.8 Looking Ahead

In April 2026, the Australian Government announced its decision to extend funding for the NACE and the NAC by 2 years, reflecting the significant achievements of these key allergy bodies. Long‐term Australian Government investment will be critical to sustain Australia's global leadership in allergy research and evidence‐based care.

Looking ahead, NACE priorities beyond 2028 include:

- Tackling complex clinical challenges through continued investment in embedded clinical research, with a strong focus on rigorous evaluation and translation to improve clinical care for everyone living with allergic disease.
- Advancing ALBI infrastructure to integrate a wider range of allergy datasets and deliver an enhanced, user‐friendly interface to support research access and use to rapidly answer questions related to the prevention, management, and treatment of allergic disease.
- Expanding the NACE living evidence collection, enabling rapid, high‐quality synthesis of emerging and existing data, supporting decision‐making and strategic partners to inform clinical guidelines and policy.
- Building capacity by identifying, training, and mentoring emerging allergy researchers to guarantee a skilled and responsive workforce to continue to address Australia's allergy burden.

With continued national support, the NACE is positioned to lead Australia into the next era of allergy research and clinical innovation, delivering lasting health and societal benefits. Goals include a reduction in allergy prevalence and anaphylaxis presentations and improved access to evidence‐based treatments across Australia's diverse geographical regions and populations.

## 4 Conclusion

As Australia's peak allergy research body, the NACE delivers essential infrastructure across the spectrum of disease—including drug, food, insect and respiratory allergies—and bridges basic science, clinical research, health services, public health, and advocacy. Through the development of research tools, resources, and enabling platforms, NACE programs accelerate allergy research and translation into improved care.

In partnership with the NAC, ASCIA and A&AA, the NACE has helped transform the national allergy landscape. Together, they have launched innovative programs to address current and emerging challenges, streamlined the collection of allergy‐related data and biospecimens, and rapidly translated research into clinical practice—ultimately improving outcomes and reducing the burden of allergic disease for Australians.

## Author Contributions

K.P.P. involved in conceptualisation; A.Y., K.P.P., J.J.K., J.M.D. and H.E. involved in writing – original draft preparation; all authors involved in writing – review and editing.

## Funding

The NACE, hosted at the MCRI, is supported by the Australian Government, Department of Health, Disability and Ageing, philanthropic donations to the MCRI and the Victorian Government's Operational Infrastructure Support Program. A.Y., T.O., D.W., E.K., L.A.G., H.E., C.J.H. and T.J. are direct employees of the NACE. K.P.P. is supported by a Melbourne Children's Clinician‐Scientist Fellowship and an NHMRC fellowship (GNT2008911). D.J.P. is supported by fellowships from The Kids Research Institute Australia (2022–2024) and Stan Perron Charitable Foundation (2025–2029). C.J.H. is also supported by a Postdoctoral Fellowship funded through the CFARCRE (NHMRC GNT2015724). J.J.K. is supported by an NHMRC fellowship (GNT1158699). J.L. has received funding from the Centre of Research Excellence in Treatable Traits in Asthma. S.V. is employed by the NAC which is supported by funding from the Australian Government, Department of Health, Disability and Ageing. M.L. has received funding for educational talks and advisory board meetings from Arrotex, CSL Behring, CSL Seqirus, GSK, Boehringer Ingelheim, Menarini, Novartis, SOBI and Stallergenes Greer. J.M.D. research is currently funded by ARC (DP240203307), NHMRC (2032605), and MRFF (2031254) as well as the NACE. In the past 3 years, on behalf of J.M.D., Q.U.T. has also received grant funding from ARC (DP210100347 and LP190100216), the Queensland Government Department of Health, and Bayer Healthcare. K.G. is CIA for the following funding: NHMRC MRFF (2022/MRF2015841 and 2024/MRF2032881); 2024/MRFF TTRA PrIDE Study; NSW Health (via University of Newcastle MIA) for Community pharmacists in NSW study; the Love Your Sister Foundation for the PAGES Study; and the National Heart Foundation for the SWiTCH Study. J.T. receives support from the NHMRC as an Emerging Leadership Fellowship. T.W. is employed by the Department of Clinical Immunology and Allergy, Incorporating the Jack Jumper Allergy Program, which is supported by funding from the Australian Government, Department of Health, Disability and Ageing.

## Ethics Statement

Individual ethics approval reference numbers are listed for studies referenced in the manuscript as applicable.

## Conflicts of Interest

K.P.P. has received research grants from Aravax, DBV Technologies, Novartis and Siolta and consultant fees from Aravax, Novartis and RAPT Therapeutics, paid to their institution, outside the submitted work and is a non‐executive Director of OmnisOva. D.E.C. is employed by DBV Technologies. Unrelated to this article, J.L. has received speaker fees and travel grants from Sanofi, Fondazione Menarini and GSK. She has been on advisory boards for Tezepelumab (Astra Zeneca). R.L.P. has received research funding from the NHMRC, National Peanut Board, Asthma Australia, Thoracic Society of Australia and New Zealand, and Allergy and Immunology Foundation of Australasia; a prize from the Stallergenes Greer Foundation; and speaker fees from Thermo Fisher Scientific, all paid to their institution outside of the submitted work. J.J.K. was a named investigator on a grant from Sanofi Regeneron for unrelated research and received a research award from the Stallergenes Greer Foundation, paid to her institution, unrelated to the current manuscript. T.W. has received research funding from the Royal Hobart Hospital Research Foundation and Wilson Foundation for works unrelated to this article. On behalf of J.M.D., Queensland University of Technology (QUT) has received a loan of pollen monitoring equipment from Swisens SA, Switzerland, services from Kenelec Australia, and an investigator‐initiated grant from Bayer Healthcare LLC, USA. J.M.D. is an inventor on patents owned by QUT; US PTO 14/311944 issued and PCT WO 2025/102125. M.D.O. reports having received grants from Government of Western Australia Department of Health and Channel 7 Telethon Trust and is a Director of ASCIA. Other authors declare no conflicts of interest for this work.

## Data Availability

This is a review article and data referenced within the text is available in the listed citations under the References section. Data generated from individual studies and trials that are underway and described briefly in the review will be made available though the NACE Allergy BioRepository (ALBI).
